# Elemental imbalance and oxidative biomarker shifts in lumbar disc degeneration

**DOI:** 10.3389/fneur.2025.1662845

**Published:** 2025-10-30

**Authors:** Damian Strojny, Roman Wojdyła, Klaudia Skóra, Martyna Hoczela, Katarzyna Wyczarska-Dziki, Mateusz Rajchel, Mateusz Miller, Dawid Sobański, Rafał Staszkiewicz, Jerzy Wieczorek, Artur Chwalba, Przemysław Rogoziński, Beniamin Oskar Grabarek

**Affiliations:** ^1^Department of Neurology, New Medical Techniques Specjalist Hospital of St. Family in Rudna Mała, Rzeszow, Poland; ^2^Collegium Medicum, WSB University, Da̧browa Górnicza, Poland; ^3^Department of Cardiology and Cardiovascular Interventions, University Hospital in Cracow, Cracow, Poland; ^4^Department of Neurological Rehabilitation, District Hospital of St. Padre Pio in Sȩdziszów Małopolski, Sȩdziszów Małopolski, Poland; ^5^Medical College Nursing Facility University of Information Technology and Managment in Rzeszow, Rzeszów, Poland; ^6^Department of Neurology, Independent Public Healthcare Institution of the Ministry of Internal Affairs and Administration in Rzeszów, Rzeszów, Poland; ^7^Department of Neurosurgery, St. Raphael Hospital, Krakow, Poland; ^8^Department of Neurosurgery, 5th Military Clinical Hospital with the SP ZOZ Polyclinic in Krakow, Krakow, Poland; ^9^Department of Histology, Cytophysiology and Embryology, Faculty of Medicine in Zabrze, Academy of Silesia in Katowice, Zabrze, Poland; ^10^Department of Agricultural and Environmental Chemistry, University of Agriculture in Krakow, Krakow, Poland; ^11^General Rehabilitation Sub-Department, Edmund Wojtyla Małopolski Hospital for Lung Diseases and Rehabilitation, Jaroszowiec, Poland; ^12^Department of Pharmacology, Faculty of Medical Sciences in Zabrze, Medical University of Silesia in Katowice, Zabrze, Poland; ^13^Dental Office, Resdent Stomatology Rogozinscy, Rzeszow, Poland; ^14^Faculty of Medicine and Health Sciences, Andrzej Frycz Modrzewski University in Cracow, Cracow, Poland

**Keywords:** intervertebral disc degeneration, ICP-OES, micronutrients, macronutrients, oxidative stress, Pfirrmann classification, lipid peroxidation, glutathione system

## Abstract

**Introduction:**

The pathogenesis of intervertebral disc degeneration (IVDD) involves multifactorial biochemical and metabolic disturbances; however, the contribution of trace elements and oxidative stress to this process remains insufficiently characterized. Understanding alterations in elemental composition and redox homeostasis within degenerated discs may reveal novel aspects of IVDD biology and potential therapeutic targets.

**Methods:**

This study analyzed lumbar intervertebral disc (IVD) tissue obtained from 200 patients undergoing microdiscectomy for lumbosacral IVDD and 100 postmortem controls without disc degeneration. The concentrations of zinc (Zn), magnesium (Mg), calcium (Ca), phosphorus (P), iron (Fe), manganese (Mn), copper (Cu), lead (Pb), sodium (Na), and potassium (K) were quantified using inductively coupled plasma optical emission spectrometry (ICP-OES). Oxidative stress status was assessed by measuring thiobarbituric acid reactive substances (TBARs; nmol MDA/mg protein), reduced glutathione (GSH; μmol/g tissue), and glutathione peroxidase (GPx; U/mg protein). Elemental concentrations were analyzed in relation to Pfirrmann degeneration grade, body mass index (BMI), and pain severity.

**Results:**

Degenerated discs exhibited significantly higher concentrations of Zn (35.10 ± 22.00 vs. 22.30 ± 14.00 mg/kg, *p* = 0.027), Mg (62,000 ± 80,000 vs. 130 ± 90 mg/kg, *p* < 0.0001), Ca (6,100 ± 13,900 vs. 1,500 ± 730 mg/kg, *p* < 0.0001), and *P* (5,000 ± 5,500 vs. 1,600 ± 1,340 mg/kg, *p* < 0.0001) compared with controls. Zn concentrations peaked in Pfirrmann grade 4 discs, while Mg was highest in grades 2 and 5 (*p* < 0.001). Ca and P levels were independent of degeneration grade but significantly elevated in obese patients (*p* < 0.001). In contrast, Mg concentrations were greatest in patients with normal BMI and declined with increasing BMI (*p* < 0.001). No significant correlations were observed between element concentrations and pain intensity (*p* > 0.05). Oxidative stress parameters indicated elevated TBARs (5.52 ± 2.52 vs. 2.63 ± 0.97, *p* < 0.0001), increased GPx activity (69.45 ± 3.92 vs. 60.25 ± 3.52, *p* = 0.027), and a non-significant reduction in GSH (*p* = 0.054). Strong positive correlations were found between *P* and Ca (*r* = 0.93, *p* < 0.001) and between K and Mn (*r* = 0.80, *p* < 0.001).

**Discussion:**

These findings reveal substantial alterations in the micro- and macronutrient composition of degenerated IVDs, particularly for Zn, Mg, Ca, and P, suggesting that metabolic dysregulation and mineral imbalance contribute to disc pathology. The observed oxidative stress profile, characterized by lipid peroxidation and compensatory GPx activation, supports the involvement of redox imbalance in IVDD progression. Together, these results underscore the interplay between mineral homeostasis, oxidative stress, and disc degeneration, providing potential avenues for biomarker development and metabolic intervention in IVDD management.

## 1 Introduction

The spine is located in the medial part of the body on the dorsal side and serves as a mobile axis for the trunk and neck (1). It is composed of 33–34 unpaired, symmetrically structured vertebrae, which are closely aligned. Between each vertebra lie intervertebral discs (IVDs) (1). A crucial element in their cross-section is the nucleus pulposus (NP), situated at the center of the IVD and surrounded by several layers of the annulus fibrosus (AF). The AF is a critical component responsible for ensuring spinal mobility and elasticity, as well as mechanical stability (2). IVD degeneration in the lumbosacral (L/S) region of the spine occurs as a result of repeated mechanical overload and increased spinal mobility (3). Predisposing factors include age, scoliosis, and trauma in the affected spinal region (3). The degenerative process is strongly associated with IVD dehydration (3). A review of the available literature indicates that IVD degeneration affects up to 90% of the general population (3). Most individuals with the condition remain asymptomatic (4). Approximately 85% of L/S IVD degeneration cases involve the L5–S1 space, with another 12% affecting the L4–L5 segment (5). Factors such as age, sex, ethnicity, occupational demands, genetic predisposition, type of physical activity, and quality of life are also associated with IVD degeneration (6). A wide range of nutrients plays a vital role in maintaining proper body function (7). These nutrients include both macronutrients and micronutrients (5). Macronutrients are minerals naturally abundant in the environment, such as nitrogen (N), chlorine (Cl), phosphorus (P), magnesium (Mg), potassium (K), sodium (Na), sulfur (S), oxygen (O), calcium (Ca), carbon (C), and hydrogen (H). These elements form integral components of enzymes, hormones, cells, and bodily fluids (9). They are essential for the normal functioning of the human body, with daily requirements exceeding 100 mg (9). Macronutrients also serve as major structural components of bones and tissues and play a critical role in water exchange and transport (10). In contrast, micronutrients are required in much smaller amounts, with daily needs of less than 100 mg (10). Key micronutrients include iron (Fe), fluorine (F), manganese (Mn), zinc (Zn), chromium (Cr), and selenium (Se) (5). While essential, excess accumulation of micronutrients in the human body can be toxic and lead to numerous adverse effects (5). Although recent research suggests that oxidative stress is a significant factor influencing IVD degeneration, current knowledge remains fragmented. Convincing evidence regarding the effectiveness of antioxidants in preventing or slowing IVD degeneration is still lacking (11). Nevertheless, maintaining relatively stable concentrations of micro- and macronutrients, including trace elements, supports enzymatic protein activity and mitigates oxidative stress (12).

Lipid peroxidation, a hallmark of oxidative damage, has been extensively documented in disc pathology (13). Thiobarbituric acid reactive substances (TBARs), generated during lipid peroxidation, have been detected at elevated levels in both animal models and human IVD tissues affected by degeneration (14). Despite these findings, the redox status of patients with degenerative disc disease remains incompletely understood. Some studies report reduced levels of enzymatic antioxidants such as superoxide dismutase (SOD) and glutathione peroxidase (GPx) in plasma and degenerated disc tissues, while others suggest that antioxidant enzyme activity may increase with age in healthy individuals, potentially as a compensatory mechanism. In patients with disc herniation or degeneration, reduced concentrations of glutathione (GSH) and glutathione reductase have also been reported, highlighting a compromised antioxidant defense system that may exacerbate tissue damage and impair disc homeostasis (14–16).

Assessing changes in micro- and macronutrient concentrations in IVDs in the context of degenerative disc disease is crucial. Such analysis can help identify enzymes and metabolites involved in the degenerative process and clarify the role of the vertebral endplate in IVD pathology (11).

Therefore, the aim of this study was to evaluate the impact of changes in the concentrations of Cu, Fe, Mn, Pb, Zn, Na, K, P, Ca, and Mg, as well as oxidative stress biomarkers, on the degenerative process of human L/S IVDs.

## 2 Material and methods

### 2.1 Ethical consideration

The study was approved by the Bioethics Committee of the State Academy of Applied Sciences in Przemyśl (approval number: 8/2024) on 01 August 2024. Written informed consent was obtained from all participants. This included consent for participation, the use of their data for analysis, and permission to publish results based on anonymized data. Participants were informed of their right to withdraw from the study at any time without consequence. The present study builds upon previous research conducted by our team and others (17).

### 2.2 Study group

The study included 200 patients (94 women and 106 men) of Caucasian ethnicity, aged 49.56 ± 15.19 years. All participants were qualified for microdiscectomy due to L/S IVD degeneration based on clinical examination, reported symptoms, and imaging findings. The mean body weight was 83.14 ± 15.83 kg, height 172.39 ± 12.31 cm, and body mass index (BMI) 26.43 ± 3.38 kg/m^2^. Patients were categorized as follows: normal weight (BMI 18.5–24.99 kg/m^2^, *n* = 43), overweight (BMI 25–29.99 kg/m^2^, *n* = 105), and obese (BMI >30 kg/m^2^, *n* = 52). All patients used standard painkillers, anti-inflammatory drugs, and muscle relaxants.

Inclusion criteria were: age ≥18 years, voluntary informed consent, isolated L/S IVD degeneration confirmed by magnetic resonance imaging (MRI), and qualification for microdiscectomy. Eligible patients reported dominant discogenic pain without sciatica, unresponsive to conservative treatment for at least 6 weeks, and had no coexisting spinal, inflammatory, or autoimmune pathologies. Pain exacerbation had to be of less than 12 weeks' duration.

Exclusion criteria included: age < 18 years, absence of consent, protrusion or sequestration of disc material on MRI, history of spinal surgery in the L/S region, or diagnosis of inflammatory diseases, autoimmune disorders, polyneuropathy, metabolic conditions, cancer, or osteoporosis. Patients with pain lasting < 6 weeks or >12 weeks were also excluded.

Each participant underwent neurological assessment by the same neurosurgery specialist at a consistent time of day. The examination included evaluation of lower limb muscle strength and tone, range of passive movement, reflex testing (patellar, Achilles, plantar, Babinski, Rossolimo), superficial and deep sensation, gait, posture, mobility, and L/S pain. Pain severity was measured using the Visual Analog Scale (VAS), where patients rated L/S pain from 0 to 10. Results were recorded in the study database.

All patients underwent preoperative MRI using a Signa Hde 1.5T (General Electric Medical System, Poland) to assess radiological changes. MRI sequences included spin echo (SE) T1, SE T1 Fluid-Attenuated Inversion Recovery (FLAIR), fast spin echo (FSE) T2, and Short Tau Inversion Recovery (STIR) in transverse and sagittal planes, with slice thicknesses of 3 mm and 4 mm. The degree of L/S IVD degeneration was independently assessed using the Pfirrmann scale by two neurosurgeons. Baseline demographic and clinical characteristics of both IVDD patients and postmortem controls are summarized in Table 1.

**Table 1:** Baseline characteristics of study participants.

**Variable**	**Study group (IVDD patients, *n* = 200)**	**Control group (postmortem, *n* = 100)**
Age, years (mean ± SD)	49.56 ± 15.19 [revision 2,209 frontiers]	37.3 ± 1.8 [revision 2,209 frontiers]
Sex, *n* (%)	94 women (47.0%), 106 men (53.0%) [revision 2,209 frontiers]	53 women (53.0%), 47 men (47.0%) [revision 2,209 frontiers]
Body weight, kg (mean ± SD)	83.14 ± 15.83 [revision 2,209 frontiers]	81.41 ± 13.15 [revision 2,209 frontiers]
Height, cm (mean ± SD)	172.39 ± 12.31 [revision 2,209 frontiers]	171.76 ± 12.32 [revision 2,209 frontiers]
BMI, kg/m^2^ (mean ± SD)	26.43 ± 3.38 [revision 2,209 frontiers]	28.19 ± 5.34 [revision 2,209 frontiers]
BMI category, *n* (%)	Normal 43 (21.5%); Overweight 105 (52.5%); Obese 52 (26.0%) [revision 2,209 frontiers]	Normal 6 (6.0%); Overweight 19 (19.0%); Obese 9 (9.0%) [revision 2,209 frontiers]
Pain (VAS, 0–10)	Recorded pre-op; used for stratified analyses (see Tables 5, 12)	n/a
MRI grading	Pfirrmann grading by two neurosurgeons (1.5 T MRI) [revision 2,209 frontiers]	Not applicable (controls histology-screened) [revision 2,209 frontiers]
Medications prior to surgery	Standard analgesics/NSAIDs/muscle relaxants in all participants [revision 2,209 frontiers]	n/a
Control inclusion	n/a	H&E confirmed no degenerative changes; age 18–45; no spinal/neoplastic/autoimmune/metabolic disease per records/family interview [revision 2,209 frontiers]

SD, standard deviation; BMI, body mass index; VAS, Visual Analog Scale for pain; MRI, magnetic resonance imaging; NSAIDs, non-steroidal anti-inflammatory drugs; H&E, hematoxylin and eosin.

### 2.3 Control group

The control group included 100 Caucasian individuals (53 women, 47 men) aged 37.3 ± 1.8 years, from whom IVDs were collected postmortem from the L/S spine. Postmortem examinations were performed within 48 h of death. Clinical data were obtained from medical records and interviews with close relatives.

Control IVDs were included following immunohistochemical staining with hematoxylin and eosin (H&E). Eligibility required age 18–45 years, no signs of IVD degeneration on histological examination, and no history of spinal, neoplastic, inflammatory, autoimmune, or metabolic disease. Exclusion criteria included age < 18 or >45 years, histological evidence of IVD degeneration, or medical history of conditions affecting disc composition or structure.

The mean body weight of the control group was 81.41 ± 13.15 kg, height 171.76 ± 12.32 cm, and BMI 28.19 ± 5.34 kg/m^2^. Normal body weight (BMI 18.5–24.99 kg/m^2^) was recorded in 6 individuals (4 women, 2 men), overweight (BMI 25–29.99 kg/m^2^) in 19 (10 women, 9 men), and obesity (BMI >30 kg/m^2^) in 9 (5 women, 4 men). Baseline demographic and clinical characteristics of IVDD patients and controls are presented in Table 1.

### 2.4 Collection of biological material for biochemical analysis: IVD tissue during microdiscectomy

IVD tissue samples were collected en bloc during microdiscectomy. The procedure involved an incision in the L/S region and muscle dissection to expose the spinal segment and canal. The sequestrated IVD fragment was removed, followed by discectomy. Blood accumulated at the surgical site was drained using a catheter, removed within 24 h post-surgery.

### 2.5 Collection of IVD tissue postmortem

During forensic autopsy or organ retrieval, the anterior surface of the L/S spine was exposed. A flat sectioning knife was used to excise IVD tissue, which was then placed in a labeled, single-use, sealed bag.

### 2.6 H&E staining

All IVD samples, whether from microdiscectomy or postmortem collection, were processed for histological evaluation with H&E staining on 5-μm paraffin sections. Staining was performed using a commercial kit (Abcam, Cambridge, MA, USA) containing hematoxylin (modified Lillie–Mayer's formulation) and eosin Y. After deparaffinization, sections were rehydrated through graded ethanol, immersed in hematoxylin for 3–5 min, rinsed, counterstained with eosin Y for 2–3 min, dehydrated, cleared in xylene, and mounted with coverslips. Slides were examined under light microscopy.

**Figure 1:**
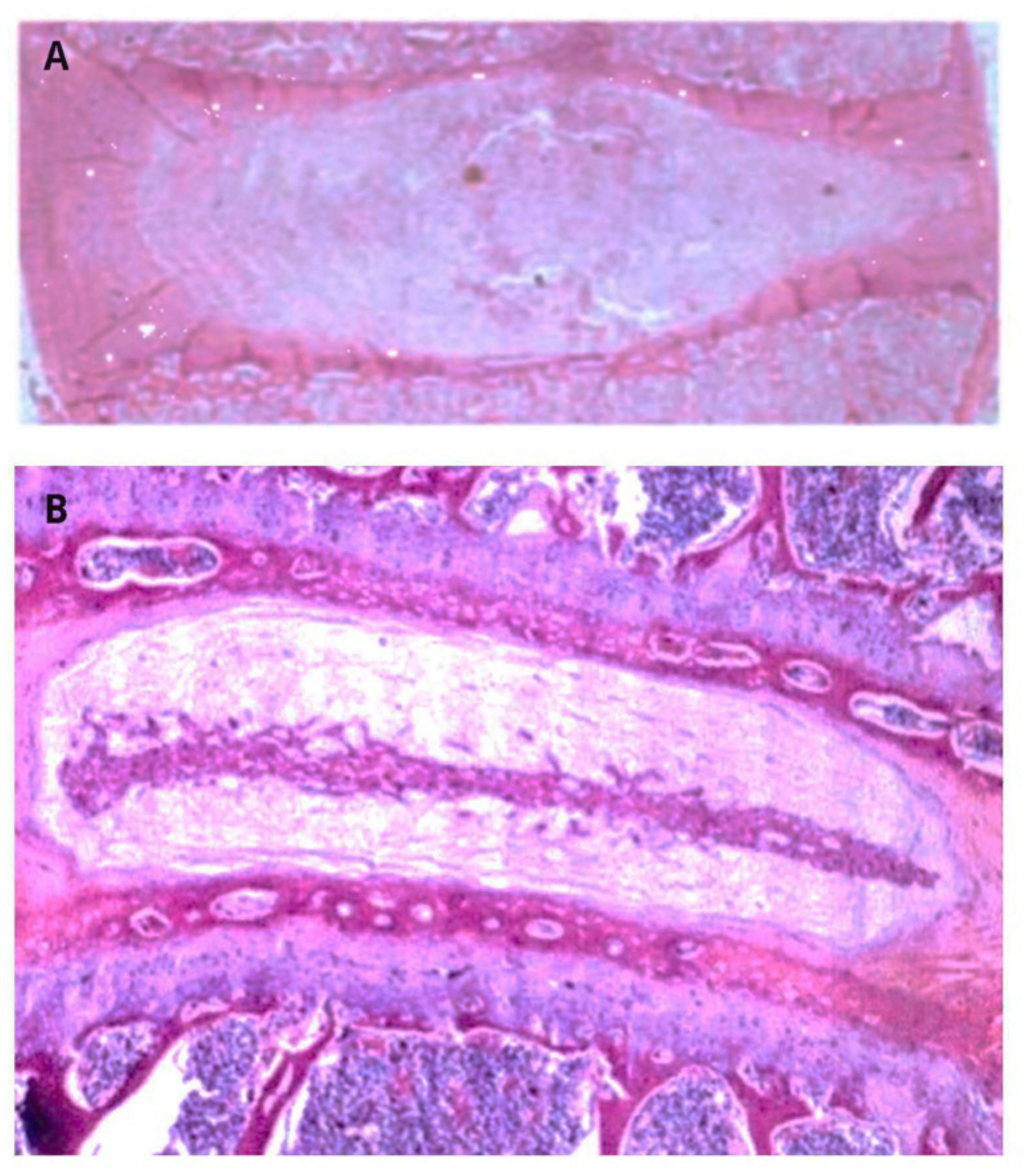
Representative H&E stained sections of human intervertebral discs. **(A)** Non-degenerate control disc showing preserved AF lamellar architecture, homogeneous NP, and absence of fissures, cell clustering, neovascularization, or endplate irregularities. **(B)** Degenerated disc demonstrating AF lamellar disorganization, fissures/clefts, clustering of NP cells with chondrocyte-like proliferation, mucoid matrix change, and irregular endplate structure. The morphological features illustrated correspond to established histopathological criteria used in validated scoring systems for human disc degeneration (18–20).

Histopathological evaluation used validated human disc scoring systems, including the Boos Histologic Degeneration Score (18), Rutges et al. classification (19), and the Orthopedic Research Society (ORS) Spine Section consensus framework (20). Degenerated discs were identified by annulus fibrosus lamellar disorganization, fissures/clefts, clustering of nucleus pulposus cells with chondrocyte-like proliferation, mucoid matrix changes, endplate irregularities, and, in some cases, neovascularization (18, 21). Control samples lacked these features. H&E was selected as the reference stain because it remains the gold standard for evaluating disc architecture and cellular morphology (18–20). Representative non-degenerate and degenerate IVDs are shown in Figure 1.

### 2.7 Determination of selected micro- and macronutrient concentrations in L/S IVD samples from study and control groups

Biochemical analyses were performed at the Department of Agricultural and Environmental Chemistry, Faculty of Agricultural and Economic Sciences, Hugo Kołataj University of Agriculture in Kraków. Inductively coupled plasma optical emission spectrometry (ICP-OES) was used for quantification. IVD samples from both groups were weighed, and 0.3–0.5 g dry mass was digested in a mixture of concentrated nitric acid (HNO3, 6 cm^3^) and hydrochloric acid (HCl, 1 cm3) of suprapur grade (Merck, Saint Louis, USA) using a Multiwave 3000 microwave system (Anton Paar, Graz, Austria). Digestion was conducted at maximum power (1,400 W) for 25 min (10-min ramp-up, 15-min sustain). Digests were filtered into 10 cm3 volumetric flasks using 1% HNO3. Element concentrations were measured with an Optima 7300 Dual View atomic emission spectrometer (Perkin Elmer). Each sample was analyzed in triplicate.

### 2.8 Measurement of lipid peroxidation products

Lipid peroxidation was quantified by thiobarbituric acid reactive substances (TBARs), following the fluorometric protocol of Asakawa and Matsushita (22). In summary, 500 μl of sample homogenate was diluted to 2 ml with phosphate-buffered saline (PBS), after which 100 μl of 100 mM butylated hydroxytoluene (BHT), 1 ml of 10% trichloroacetic acid (TCA), 500 μl of 5 mM ethylenediaminetetraacetic acid (EDTA), and 250 μl of 8% sodium dodecyl sulfate (SDS) were sequentially added. Subsequently, 750 μl of 0.6% thiobarbituric acid (TBA) was added, and the mixture was incubated at 90 °C for 1 h. After cooling to room temperature, samples were centrifuged at 1,690 × g for 10 min using a benchtop centrifuge (TOMY LC-200, TOMY Digital Biology Co., Ltd., Tokyo, Japan). The fluorescence of the resulting supernatant was read at excitation/emission wavelengths of 515/553 nm using a Varioskan Flash Multimode Microplate Reader (Thermo Fisher Scientific, Waltham, MA, USA). Calibration was performed using 1,1,3,3-tetraethoxypropane as a standard.

### 2.9 Assessment of GSH levels

Total GSH concentration was determined based on the DTNB–glutathione reductase recycling assay as described by Anderson (23). This method employs 5,5′-dithiobis(2-nitrobenzoic acid) (DTNB), which reacts with thiol groups to produce a yellow chromophore. Specifically, 50 μl of the sample supernatant was mixed with 200 μl of a reaction solution containing 0.4 mM nicotinamide adenine dinucleotide phosphate (NADPH) and 1.2 U/ml glutathione reductase in 0.01 M sodium phosphate buffer supplemented with 0.05 mM ethylenediaminetetraacetic acid (EDTA). After incubation at 30 °C for 2 min, 20 μl of DTNB was added. Absorbance was measured at 412 nm at 30-s intervals for 3 min using a Varioskan Flash Multimode Microplate Reader (Thermo Fisher Scientific, Waltham, MA, USA). GSH concentrations were calculated by comparing the slopes with a standard curve generated from known GSH concentrations and expressed as nmol/mg protein.

### 2.10 Measurement of glutathione peroxidase (GPx) activity

GPx enzymatic activity was measured using a spectrophotometric approach in accordance with the method of Takahashi (24). A 20 μl aliquot of the sample supernatant was incubated with 185 μl of a reaction mixture containing 25 mM reduced GSH, 0.2 mM NADPH, and 1.25 mM EDTA in 0.25 M Tris-HCl buffer. An additional 25 μl of glutathione reductase (10 U/ml) was then added. After 2 min of incubation at 37 °C, the reaction was initiated by adding 20 μl of 1.75 mM tert-butyl hydroperoxide (t-BuOOH). The decrease in absorbance, corresponding to NADPH oxidation, was monitored at 340 nm every 15 s for a total of 10 min using the Varioskan Flash Multimode Microplate Reader (Thermo Fisher Scientific, Waltham, MA, USA). GPx activity was calculated using the NADPH extinction coefficient (ε = 2.57 mM^−1^cm^−1^) and expressed as μmol NADPH oxidized per minute per mg of protein.

### 2.11 Statistical analysis

Statistical analysis was performed using Statplus software (AnalystSoft Inc., Brandon, FL, USA), with the level of significance set at *p* < 0.05. Initially, numerical data were tested for normal distribution using the Shapiro–Wilk test. The outcome of this test determined whether parametric methods were applied in subsequent analyses.

For comparisons between two groups, the independent Student's *t*-test was used. For comparisons among three or more groups, one-way analysis of variance (ANOVA) was applied, with homogeneity of variances verified using Levene's test. When ANOVA revealed statistically significant differences, *post-hoc* analysis was performed using Scheffé's test.

Relationships between the concentrations of micro- and macronutrients in IVD samples from the study and control groups were assessed using Pearson's correlation analysis. Data are presented as mean ± standard deviation (SD) with a 95% confidence interval (CI). Larger SD values in some datasets reflect biological variability within the cohort rather than violations of normality assumptions.

## 3 Results

### 3.1 Concentrations of micro- and macronutrients in degenerated and non-degenerated L/S IVDs

In the initial phase of this doctoral research, the concentrations of micro- and macronutrients were compared between the study and control samples (Table 2).

**Table 2:** Concentrations of micro- and macronutrients in IVDs from study and control groups.

**Micro-/macronutrient**	**Group**	**Concentration [mg/kg-1 d.m.]**	**95% confidence interval**	***p* (student's *t*-test)**
Cu	Study	2.45 ± 2.71	2.00–2.90	0.090
	Control	3.64 ± 2.14	2.70–4.60	
Fe	Study	145.24 ± 56.73	134.00–156.00	0.290
	Control	130.70 ± 50.38	106.00–155.00	
Mn	Study	0.50 ± 0.45	0.38–0.60	0.440
	Control	0.42 ± 0.40	0.28–0.50	
Pb	Study	0.92 ± 0.65	0.78–1.06	0.070
	Control	0.62 ± 0.28	0.50–0.75	
Zn	Study	35.10 ± 22.00	28.00–42.00	0.027
	Control	22.30 ± 14.00	15.00–30.00	
Na	Study	14200.00 ± 10100.00	12100.00–16100.00	0.690
	Control	13300.00 ± 1970.00	12200.00–14200.00	
Mg	Study	62000.00 ± 80000.00	46000.00–77000.00	< 0.0001
	Control	130.00 ± 90.00	80.00–165.00	
K	Study	295.00 ± 370.00	215.00–365.00	0.630
	Control	335.00 ± 150.00	265.00–405.00	
Ca	Study	6100.00 ± 13900.00	3300.00–8700.00	< 0.0001
	Control	1500.00 ± 730.00	1110.00–1790.00	
P	Study	5000.00 ± 5500.00	3900.00–6100.00	< 0.0001
	Control	1600.00 ± 1340.00	930.00–2200.00	

Cu, copper; Fe, iron; Mn, manganese; Pb, lead; Zn, zinc; Na, sodium; Mg, magnesium; K, potassium; Ca, calcium; P, phosphorus; d.m., dry mass; p, statistical significance; 95%CL, 95% confidence interval. Data are presented as mean ± standard deviation.

Statistical analysis revealed that Zn concentrations were significantly higher in the study group compared to the control group (35.10 mg/kg dry mass (d.m.) ± 22.00 mg/kg d.m. vs. 22.30 mg/kg d.m. ± 14.00 mg/kg d.m.; Table 2; *p* < 0.05). Similarly, Mg levels were significantly elevated in the study samples compared to the controls (62,000.00 mg/kg d.m. ± 80,000.00 mg/kg d.m. vs. 130.00 mg/kg d.m. ± 90.00 mg/kg d.m.; Table 2; *p* < 0.05).

Additionally, *p* concentrations were significantly higher in the study samples than in the control group (5,000.00 mg/kg d.m. ± 5,500.00 mg/kg d.m. vs. 1,600.00 mg/kg d.m. ± 1,340.00 mg/kg d.m.; Table 2; *p* < 0.05). Ca levels were also significantly elevated in the study samples compared to controls (6,100.00 mg/kg d.m. ± 13,900.00 mg/kg d.m. vs. 1,500.00 mg/kg d.m. ± 730.00 mg/kg d.m.; Table 2; *p* < 0.05). No statistically significant differences were observed for the remaining micro- and macronutrients (Table 2; *p* > 0.05).

### 3.2 Concentration of micro- and macronutrients in degenerated and control L/S IVDs by gender

The study next evaluated whether concentrations of micro- and macronutrients in L/S IVDs from the study group differed according to patient gender (Table 3). Statistical analysis showed that only Mn concentrations differed significantly, being higher in women than in men (0.58 mg/kg d.m. ± 0.51 mg/kg d.m. vs. 0.42 mg/kg d.m. ± 0.34 mg/kg d.m.; Table 3; *p* < 0.05). No other statistically significant gender-related differences were observed (Table 3; *p* > 0.05).

**Table 3:** Concentration of micro- and macronutrients in IVDs obtained from women and men in the study group.

**Micro-/macronutrient**	**Gender**	**Concentration [mg/kg-1 d.m.]**	**95% confidence interval**	***p* (student's *t*-test)**
Cu	Women	2.63 ± 2.89	1.65–3.61	0.770
	Men	2.75 ± 2.87	2.00–3.50	
Fe	Women	155.12 ± 48.97	136.80–173.45	0.402
	Men	141.45 ± 62.15	125.50–157.45	
Mn	Women	0.58 ± 0.51	0.42–0.74	0.045
	Men	0.42 ± 0.34	0.33–0.51	
Pb	Women	0.93 ± 0.78	0.69–1.17	0.890
	Men	0.95 ± 0.61	0.78–1.03	
Zn	Women	35.12 ± 24.35	27.12–43.11	0.550
	Men	30.85 ± 19.45	25.89–35.81	
Na	Women	13752.63 ± 10359.12	10595.45–16910.23	0.741
	Men	14425.89 ± 9917.45	11985.50–16865.42	
Mg	Women	61523.12 ± 79000.12	38000.45–85000.12	0.975
	Men	6885.89 ± 81765.35	41000.45–83000.65	
K	Women	355.87 ± 491.12	205.12–505.12	0.137
	Men	248.12 ± 252.63	181.12–315.89	
Ca	Women	5201.45 ± 13799.65	970.45–9500.12	0.627
	Men	6599.78 ± 14002.78	3000.12–10300.65	
P	Women	4298.45 ± 512.85	2600.45–6050.12	0.312
	Men	5435.65 ± 435.22	4050.45–6800.65	

Cu, copper; Fe, iron; Mn, manganese; Pb, lead; Zn, zinc; Na, sodium; Mg, magnesium; K, potassium; Ca, calcium; P, phosphorus; d.m., dry mass; p, statistical significance; 95%CL, 95% confidence interval. Data are presented as mean ± standard deviation.

In the control group (Table 4), Fe concentrations were significantly higher in women compared to men (163.30 mg/kg d.m. ± 67.93 mg/kg d.m. vs. 111.96 mg/kg d.m. ± 57.16 mg/kg d.m.; Table 4; *p* < 0.05). Similarly, Zn concentrations were significantly higher in women than in men (31.43 mg/kg d.m. ± 17.26 mg/kg d.m. vs. 16.15 mg/kg d.m. ± 6.60 mg/kg d.m.; Table 4; *p* < 0.05).

**Table 4:** Concentration of micro- and macronutrients in IVDs obtained from women and men in the control group.

**Micro-/macronutrient**	**Gender**	**Concentration [mg/kg-1 d.m.]**	**95% confidence interval**	***p* (student's *t*-test)**
Cu	Women	3.51 ± 1.72	2.12–4.81	0.510
	Men	4.24 ± 2.72	2.58–5.90	
Fe	Women	163.30 ± 67.93	105.70–220.10	0.027
	Men	113.96 ± 57.16	97.78–129.24	
Mn	Women	0.54 ± 0.28	0.33–0.75	0.063
	Men	0.33 ± 0.21	0.20–0.45	
Pb	Women	0.62 ± 0.34	0.35–0.89	0,0.516
	Men	0.68 ± 0.30	0.49–0.87	
Zn	Women	31.43 ± 17.26	17.08–45.78	0.014
	Men	16.15 ± 6.60	12.01–20.28	
Na	Women	13788.25 ± 2280.78	11889.83–15686.67	0.413
	Men	13121.92 ± 1791.95	11989.71–14254.11	
Mg	Women	129.57 ± 82.78	61.27–199.85	0.765
	Men	118.83 ± 98.28	57.03–180.64	
K	Women	266.99 ± 121.19	166.58–367.49	0.091
	Men	377.53 ± 148.89	283.56–471.59	
Ca	Women	1776.50 ± 1072.99	888.77–2674.42	0.061
	Men	1236.42 ± 248.76	1084.72–1388.22	
P	Women	1796.13 ± 1400.77	625.89–2966.46	0.487
	Men	1377.25 ± 1330.38	538.32–2216.28	

Cu, copper; Fe, iron; Mn, manganese; Pb, lead; Zn, zinc; Na, sodium; Mg, magnesium; K, potassium; Ca, calcium; P, phosphorus; d.m., dry mass; p, statistical significance; 95%CL, 95% confidence interval. Data are presented as mean ± standard deviation.

### 3.3 Concentration of micro- and macronutrients in IVDs based on Pfirrmann degeneration grade

Micro- and macronutrient concentrations were further analyzed according to Pfirrmann degeneration grade (Table 5). No consistent trend was observed regarding which grade corresponded to the highest or lowest element concentrations (Table 5).

**Table 5:** Concentration of micro- and macronutrients based on the degeneration grade of IVDs.

**Mikro-/makroelement**	**Pfirrmann's level**	**Concentration [mg/kg d.m.]**	**95%CL**	***p* ANOVA (test *post-hoc*)**
Cu	2	2.45 ± 1.92	1.28–3.62	0.135
	3	2.48 ± 2.55	1.50–3.46	
	4	3.30 ± 3.60	2.20–4.40	
	5	1.12 ± 0.75	0.55–1.70	
Fe	2	170.10 ± 65.25	105.00–215.00	0.044 (2 vs. 4)
	3	162.10 ± 55.35	145.00–180.00	
	4	128.30 ± 53.70	110.00–145.00	
	5	142.80 ± 50.22	115.00–170.00	
Mn	2	0.29 ± 0.32	0.05–0.62	0.036 (2 vs. 3; 2 vs. 5)
	3	0.65 ± 0.58	0.42–0.88	
	4	0.51 ± 0.37	0.40–0.62	
	5	0.32 ± 0.30	0.10–0.55	
Pb	2	1.18 ± 0.95	0.60–1.80	0.085
	3	0.82 ± 0.70	0.55–1.10	
	4	0.85 ± 0.55	0.70–1.00	
	5	1.25 ± 0.98	0.65–1.90	
Zn	2	37.50 ± 28.00	18.00–57.00	0.615
	3	29.50 ± 20.50	22.00–37.00	
	4	44.50 ± 22.20	30.00–50.00	
	5	27.90 ± 18.30	15.00–40.00	
Na	2	24000.00 ± 18500.00	12500.00–35500.00	0.002 (2 vs. 4; 2 vs. 5)
	3	15000.00 ± 9800.00	11000.00–18500.00	
	4	12000.00 ± 6400.00	10500.00–13700.00	
	5	11300.00 ± 6500.00	6900.00–15600.00	
Mg	2	180000.00 ± 60000.00	140000.00–220000.00	< 0.001 (2 vs. 3; 2 vs. 4; 3 vs. 5)
	3	45000.00 ± 70500.00	20000.00–70000.00	
	4	28000.00 ± 56000.00	11000.00–45000.00	
	5	132000.00 ± 65000.00	88000.00–176000.00	
K	2	280.00 ± 305.00	80.00–470.00	0.353
	3	380.00 ± 570.00	180.00–590.00	
	4	235.00 ± 105.00	175.00–300.00	
	5	280.00 ± 280.00	90.00–460.00	
Ca	2	1220.00 ± 980.00	600.00–1840.00	0.498
	3	8150.00 ± 19600.00	1200.00–15100.00	
	4	6100.00 ± 11300.00	2850.00–9400.00	
	5	4540.00 ± 11400.00	3080.00–12200.00	
P	2	3700.00 ± 2750.00	2000.00–5400.00	0.573
	3	5820.00 ± 7600.00	3100.00–8600.00	
	4	4990.00 ± 4350.00	3700.00–6200.00	
	5	3750.00 ± 4610.00	650.00–6850.00	

Cu, copper; Fe, iron; Mn, manganese; Pb, lead; Zn, zinc; Na, sodium; Mg, magnesium; K, potassium; Ca, calcium; P, phosphorus; d.m., dry mass; p, statistical significance; 95%CL, 95% confidence interval. Data are presented as mean ± standard deviation.

However, one-way ANOVA revealed that the concentrations of Fe, Mn, Na, and Mg varied significantly with the degeneration grade (*p* < 0.05). *Post hoc* analysis showed statistically significant differences in Fe concentrations between grades 2 and 4 (*p* < 0.05); Mn concentrations between grades 2 and 3, and 2 and 5 (*p* < 0.05); Na concentrations between grades 2 and 4, and 2 and 5 (*p* < 0.05); and Mg concentrations between grades 2 and 3, 2 and 4, and 3 and 5 (*p* < 0.05) (Table 5).

### 3.4 Concentration of micro- and macronutrients in L/S IVDs based on pain intensity

The study then assessed whether micro- and macronutrient concentrations were influenced by patient-reported pain intensity (Table 6). One-way ANOVA revealed no statistically significant differences in concentrations across pain intensity categories (Table 6; *p* > 0.05).

**Table 6:** Concentration of micro- and macronutrients in L/S IVDs based on pain intensity.

**Micro-/macronutrient**	**Pain intensity**								***p*-value**
	**3**	**4**	**5**	**6**	**7**	**8**	**9**	**10**	
Cu (mg/kg d.m.)	3.12 ± 2.90 (1.75–4.49)	4.21 ± 3.15 (2.60–5.82)	2.98 ± 1.86 (2.02–3.94)	1.45 ± 0.95 (0.91–1.99)	2.12 ± 1.76 (1.19–3.05)	1.78 ± 0.98 (1.22–2.34)	2.45 ± 1.23 (1.78–3.12)	1.20 ± 0.50 (0.87–1.53)	0.158
Fe (mg/kg d.m.)	153.45 ± 65.34 (120.78–186.12)	148.22 ± 58.90 (118.64–177.80)	139.50 ± 51.11 (114.45–164.55)	160.33 ± 62.45 (130.82–189.84)	175.50 ± 72.30 (138.71–212.29)	142.15 ± 58.45 (112.96–171.34)	138.10 ± 49.78 (115.09–161.11)	128.25 ± 40.85 (108.74–147.76)	0.842
Mn (mg/kg d.m.)	0.42 ± 0.30 (0.27–0.57)	0.50 ± 0.33 (0.32–0.68)	0.38 ± 0.27 (0.23–0.53)	0.55 ± 0.31 (0.38–0.72)	0.65 ± 0.42 (0.40–0.90)	0.45 ± 0.25 (0.30–0.60)	0.35 ± 0.22 (0.24–0.46)	0.30 ± 0.20 (0.21–0.39)	0.092
Pb (mg/kg d.m.)	0.85 ± 0.50 (0.58–1.12)	1.10 ± 0.62 (0.76–1.44)	1.15 ± 0.55 (0.84–1.46)	0.95 ± 0.48 (0.67–1.23)	0.90 ± 0.40 (0.67–1.13)	1.05 ± 0.65 (0.70–1.40)	0.92 ± 0.38 (0.73–1.11)	0.80 ± 0.30 (0.65–0.95)	0.365
Zn (mg/kg d.m.)	25.00 ± 21.00 (15.50–34.50)	30.45 ± 18.50 (21.82–39.08)	35.20 ± 22.80 (23.80–46.60)	28.90 ± 20.10 (19.46–38.34)	33.75 ± 15.90 (26.49–41.01)	30.80 ± 14.50 (24.94–36.66)	22.15 ± 10.50 (17.03–27.27)	18.90 ± 9.20 (14.73–23.07)	0.045
Na (mg/kg d.m.)	13,550 ± 10,500 (8,897–18,203)	14,800 ± 9,900 (10,261–19,339)	12,550 ± 8,700 (8,717–16,383)	11,200 ± 7,600 (7,593–14,807)	16,500 ± 9,400 (11,623–21,377)	17,450 ± 11,000 (11,798–23,102)	15,900 ± 9,700 (11,045–20,755)	14,300 ± 8,800 (9,657–18,943)	0.039
Mg (mg/kg d.m.)	10,500 ± 39,000 (0–25,116)	42,000 ± 76,000 (0–86,304)	38,000 ± 74,000 (3,599–73,355)	101,000 ± 75,000 (65,078–144,324)	69,000 ± 90,000 (0–164,064)	15,2000 ± 63,000 (104,342–194,638)	139,000 ± 60,000 (0–285,632)	130,000 ± 49,000 (51,931–20,8019)	0.091
K (mg/kg d.m.)	200.00 ± 180.00 (128.32–271.68)	210.00 ± 100.00 (155.78–264.22)	300.00 ± 250.00 (185.54–414.46)	370.00 ± 300.00 (230.55–509.45)	640.00 ± 1200.00 (0–1864.87)	340.00 ± 400.00 (131.78–548.22)	150.00 ± 30.00 (130.00–170.00)	140.00 ± 25.00 (120.00–160.00)	0.412
Ca (mg/kg d.m.)	5,200 ± 8,000 (2,200–8,200)	8,500 ± 17,000 (0–18,116)	9,600 ± 18,000 (1,496–18,112)	3,200 ± 9,000 (0–8,044)	14,000 ± 31,000 (0–45,999)	1,000 ± 600 (570–1,430)	1,700 ± 1,300 (570–2,830)	800 ± 600 (400–1,200)	0.521
P (mg/kg d.m.)	5,200 ± 4,000 (3,547–6,853)	5,100 ± 6,200 (1,560–8,640)	6,800 ± 7,200 (3,484–10,116)	3,700 ± 3,800 (1,721–5,679)	7,700 ± 11,000 (0–18,960)	3,200 ± 1,300 (2,251–4,100)	2,900 ± 1,100 (135–4,665)	2,000 ± 1,200 (171–3,830)	0.458

Cu, copper; Fe, iron; Mn, manganese; Pb, lead; Zn, zinc; Na, sodium; Mg, magnesium; K, potassium; Ca, calcium; P, phosphorus; d.m., dry mass; p, statistical significance; 95%CL, 95% confidence interval. Data are presented as mean ± standard deviation (95%Cl).

### 3.5 Concentration of micro- and macronutrients in degenerated L/S IVDs based on patients' BMI

The study further examined changes in the concentrations of micro- and macronutrients in degenerated IVDs depending on the BMI of the patients (Table 7). Statistically significant differences were observed in the concentrations of Mg, Ca, and *p* across BMI categories (*p* < 0.05). Therefore, *post-hoc* analysis was conducted following the ANOVA.

**Table 7:** Concentration of micro- and macronutrients in degenerated IVDs based on patients' BMI.

**Micro-/macronutrient**	**Normal**	**Overweight**	**Obesity**	***p*-value**
Cu (mg/kg d.m.)	2.15 ± 1.80 (1.38–2.92)	3.05 ± 3.20 (2.15–3.95)	2.75 ± 2.90 (1.72–3.78)	0.122
Fe (mg/kg d.m.)	140.34 ± 54.72 (116.68–163.99)	148.69 ± 58.11 (132.83–164.55)	168.42 ± 57.93 (144.42–192.42)	0.182
Mn (mg/kg d.m.)	0.32 ± 0.30 (0.18–0.46)	0.52 ± 0.53 (0.38–0.66)	0.62 ± 0.25 (0.51–0.73)	0.053
Pb (mg/kg d.m.)	1.05 ± 0.66 (0.76–1.34)	1.00 ± 0.75 (0.79–1.21)	0.90 ± 0.61 (0.66–1.14)	0.300
Zn (mg/kg d.m.)	34.18 ± 21.99 (24.67–43.69)	31.94 ± 22.63 (21.76–42.11)	30.77 ± 19.51 (25.05–36.49)	0.815
Na (mg/kg d.m.)	15,475 ± 12,669 (9,564–21,386)	14,621 ± 9,863 (11,929–17,313)	12,218 ± 6,202 (9,765–14,672)	0.750
Mg (mg/kg d.m.)	107,925 ± 75,718 (75,183–140,668)	72,636 ± 84,700 (49,518–95,755)	360 ± 337 (226–494)	< 0.001 (overweight vs. obesity; obesity vs. normal)
K (mg/kg d.m.)	260 ± 281 (138–382)	450 ± 474 (320–580)	210 ± 112 (170–250)	0.250
Ca (mg/kg d.m.)	940 ± 712 (636–1,244)	4,151 ± 7,582 (2,282–6,020)	14,060 ± 23,427 (4,893–23,227)	< 0.001 (overweight vs. obesity; obesity vs. normal)
P (mg/kg d.m.)	2,708 ± 1,394 (2,105–3,311)	4,212 ± 3,255 (3,324–5,100)	8,406 ± 8,793 (4,928–11,884)	< 0.001 (overweight vs. obesity; obesity vs. normal)

Cu, copper; Fe, iron; Mn, manganese; Pb, lead; Zn, zinc; Na, sodium; Mg, magnesium; K, potassium; Ca, calcium; P, phosphorus; d.m., dry mass; p, statistical significance; 95%CL, 95% confidence interval. Data are presented as mean ± standard deviation (95%Cl).

The highest concentration of Mg was observed in IVDs from patients with a normal BMI, while the lowest was found in IVDs from obese patients (Table 7; *p* < 0.05). Conversely, the highest concentration of Ca was recorded in IVDs from obese patients, while the lowest was found in samples from patients with a normal BMI (Table 7; *p* < 0.05). Additionally, the highest concentration of *p* was noted in IVDs from overweight patients, with the lowest concentration observed in samples from obese patients (Table 7; *p* < 0.05).

### 3.6 Correlation between micro- and macronutrient concentrations in degenerated L/S IVDs

Pearson's correlation analysis identified 15 statistically significant relationships among micro- and macronutrients in the study group. The strongest correlations were observed between K and Mn (*r* = 0.80; *p* < 0.001) and between *p* and Ca (*r* = 0.93; *p* < 0.001). Detailed results are presented in Table 8.

**Table 8:** Correlation analysis between micro- and macronutrient concentrations in study samples.

**Comparison**	**Correlation coefficient**	***p***
K vs. Mn	0.80	< 0.001
Mg vs. Na	0.47	< 0.001
P vs. Ca	0.93	< 0.001
Zn vs. Cu	0.46	< 0.001
Zn vs. Pb	0.48	< 0.001
Na vs. Cu	0.33	0.002
Mg vs. Pb	0.31	0.003
P vs. Zn	0.30	0.003
Mg vs. Mn	−0.28	0.006
Ca vs. Mg	−0.28	0.006
Mg vs. Fe	0.27	0.008
Ca vs. Zn	0.25	0.014
Na vs. Fe	0.25	0.014
Ca vs. Na	−0.24	0.021
P vs. Mg	−0.23	0.022

Cu, copper; Fe, iron; Mn, manganese; Pb, lead; Zn, zinc; Na, sodium; Mg, magnesium; K, potassium; Ca, calcium; P, phosphorus—statistical significance.

### 3.7 Correlation between micro- and macronutrient concentrations in control L/S IVDs

In the control group, Pearson's correlation analysis identified seven statistically significant relationships. The strongest correlations were observed between Mg and Cu (*r* = −0.85; *p* < 0.001), Mg and Mn (*r* = 0.72; *p* = 0.002), and Mn and Cu (*r* = −0.70; *p* < 0.05). Detailed results are presented in Table 9.

**Table 9:** Correlation analysis between micro- and macronutrient concentrations in control samples.

**Comparison**	**Correlation coefficient**	***p***
Mg vs. Cu	−0.87	< 0.001
Mg vs. Mn	0.74	0.002
Mn vs. Cu	−0.72	0.003
Pb vs. Cu	0.66	0.002
Mn vs. Fe	0.65	0.003
Na vs. Pb	0.51	0.028
Mg vs. Pb	−0.49	0.037
K vs. Fe	−0.46	0.050
P vs. Mg	0.43	0.063
P vs. Zn	0.40	0.083

Cu, copper; Fe, iron; Mn, manganese; Pb, lead; Zn, zinc; Na, sodium; Mg, magnesium; K, potassium; Ca, calcium; P, phosphorus—statistical significance.

### 3.8 Oxidative stress marker profiles in IVDs degeneration: association with pain, degeneration grade, sex, and BMI

In this study, the oxidative stress markers TBARs, GSH, and GPx were evaluated in relation to clinical and demographic variables, including pain intensity, degeneration grade, sex, and BMI (Tables 10–13). TBARs levels were significantly elevated in degenerated IVD tissues compared to controls (5.52 ± 2.52 vs. 2.63 ± 0.97; *p* < 0.0001), with female patients demonstrating a more pronounced increase. GPx activity was also significantly higher in the study group than in the controls (69.45 ± 3.92 vs. 60.25 ± 3.52; *p* = 0.0267), particularly among women, suggesting an enhanced enzymatic antioxidant response. In contrast, GSH levels displayed a decreasing trend, although the difference did not reach statistical significance (*p* = 0.0539).

**Table 10:** Comparison of oxidative stress markers (TBARs, GSH, GPx) between study and control groups, stratified by sex.

**Parameter**	**Subgroup**	**Study group (mean ±SD)**	**Control group (mean ±SD)**	***p*-value**
TBARs		5.52 ± 2.52	2.63 ± 0.97	0.0000
	Females	4.83 ± 1.02	2.41 ± 0.93	0.0255^a^
	Males	3.93 ± 1.75	2.88 ± 0.97	0.0148^b^
GSH		0.35 ± 0.28	0.43 ± 0.39	0.0539
	Females	0.37 ± 0.31	0.44 ± 0.41	0.4865^a^
	Males	0.39 ± 0.34	0.43 ± 0.37	0.9489^b^
GPx		69.45 ± 3.92	60.25 ± 3.52	0.0267
	Females	68.40 ± 4.64	59.42 ± 3.05	0.0307^a^
	Males	61.13 ± 4.49	61.17 ± 4.38	0.7964^b^

^a^*p*-value for study group.

^b^*p*-value for control group.

**Table 11:** Comparison of oxidative stress markers (TBARs, GSH, GPx) between study and control groups stratified by BMI category.

**Parameter**	**Subgroup**	**Study group (mean ±SD)**	**Control group (mean ±SD)**	***p*-value**
TBARs	Normal BMI	5.12 ± 0.90	2.60 ± 0.90	0.3990^a^
	Overweight	6.13 ± 0.89	2.46 ± 0.95	0.2936^b^
	Obese	5.34 ± 0.69	2.82 ± 1.04	
GSH	Normal BMI	0.30 ± 0.28	0.32 ± 0.46	0.1259^a^
	Overweight	0.35 ± 0.28	0.47 ± 0.35	0.1762^b^
	Obese	0.40 ± 0.26	0.49 ± 0.35	
GPx	Normal BMI	73.08 ± 7.32	56.43 ± 2.67	0.0267^a^
	Overweight	66.14 ± 3.67	63.69 ± 7.29	0.4916^b^
	Obese	68.95 ± 3.35	60.08 ±9.43	

^a^*p*-value for study group.

^b^*p*-value for control group.

**Table 12:** Comparison of oxidative stress markers (TBARs, GSH, GPx) in the study group according to Pfirrmann grade.

**Parametr**	**Pfirrmann grade**	**Study group (mean ±SD)**	***p*-value (ANOVA)**
TBARs	Grade 2	3.7 ± 1.21	0.0402
	Grade 3	5.07 ± 165	
	Grade 4	6.28 ± 1.41	
	Grade 5	6.85± 1.48	
GSH	Grade 2	0.55 ± 0.28	0.0451
	Grade 3	0.40 ± 0.28	
	Grade 4	0.25 ± 0.27	
	Grade 5	0.21 ± 0.27	
GPx	Grade 2	75.86 ± 3.82	0.0410
	Grade 3	68.98 ± 3.24	
	Grade 4	61.01 ± 3.11	
	Grade 5	68.87 ± 3.67	

**Table 13:** Comparison of oxidative stress markers (TBARs, GSH, GPx) in the study group according to pain level.

**VAS**	**TBARs (mean ±SD)**	**GSH (mean ±SD)**	**GPx (mean ±SD)**
3	3.88 ± 0.21	0.53 ± 0.03	73.02 ± 4.14
4	4.41 ± 0.21	0.47 ± 0.03	71.57 ± 4.14
5	4.94 ± 0.21	0.41 ± 0.03	70.13 ± 4.14
6	5.47 ± 0.21	0.35 ± 0.03	68.68 ± 4.14
7	6.01 ± 0.21	0.29 ± 0.03	67.23 ± 4.14
8	6.54 ± 0.21	0.24 ± 0.03	65.79 ± 4.14
9	7.07 ± 0.21	0.18 ± 0.03	64.34 ± 4.14
10	7.61 ± 0.21	0.12 ± 0.03	62.89 ± 4.14
*p*-value (ANOVA)	0.0148	0.0255	0.3844

When stratified by BMI, individuals with normal weight exhibited the highest GPx activity (73.08 ± 7.32), which may indicate a more robust antioxidant defense, whereas TBARs were most elevated in overweight patients, reflecting heightened oxidative damage. Interestingly, GSH levels increased slightly with higher BMI, which may represent a compensatory mechanism in response to chronic oxidative stress. Analysis based on Pfirrmann degeneration grade demonstrated progressive increases in TBARs (from 3.7 ± 1.21 in grade 2 to 6.85 ± 1.48 in grade 5; *p* = 0.0402) accompanied by significant reductions in GSH (from 0.55 ± 0.28 to 0.21 ± 0.27; *p* = 0.0451), consistent with an imbalance in redox homeostasis during disc degeneration. GPx activity declined from grade 2 to grade 4 but rose again at grade 5 (*p* = 0.0410), suggesting the presence of a late-stage compensatory antioxidant response.

## 4 Discussion

It has been confirmed that IVD degeneration is driven not only by local changes in neurotrophic factor concentrations, the formation of new nerve endings, and their infiltration into the IVD, but also by an imbalance in the extracellular matrix (ECM) between anabolic and catabolic enzyme activity (25, 26). A particularly important group of proteolytic enzymes directly associated with IVD degeneration are matrix metalloproteinases (MMPs), whose enzymatic activity depends on Zn (27). Moreover, the concentrations of other trace elements, such as Pb and Cd, provide information on environmental exposure, as these elements tend to accumulate in the analyzed tissues (28, 29). Therefore, profiling the concentrations of micro- and macronutrients in degenerated IVDs provides valuable insights into the metabolic changes accompanying degeneration (28, 29).

This study aimed to assess changes in the concentrations of Cu, Fe, Mn, Pb, Zn, Na, K, P, Ca, and Mg in degenerated and non-degenerated IVDs from the L/S region. Human IVDs were obtained from patients with degenerative disc disease undergoing microdiscectomy and from deceased individuals during forensic examinations or organ retrieval procedures. While there is a disparity in the size of the study group, comprising 200 patients diagnosed with degenerative disease of the L/S IVDs, and the control group, comprising 100 individuals whose L/S IVDs were obtained postmortem, it should be noted that human control samples can be appropriately obtained postmortem. This discrepancy in group sizes reflects the limited availability of non-degenerated human IVDs, as the number of organ retrievals and forensic examinations yielding such tissues is significantly lower than the number of microdiscectomy procedures. Furthermore, only L/S IVDs were used as control material, and not all postmortem samples were ultimately classified as control material due to exclusions based on histopathological assessments, including H&E staining.

A review of the available literature on IVD degenerative disease and changes in micro- and macronutrient concentration patterns in IVDs indicates that relatively few studies have been conducted on human material (25, 28, 29). Animal models are far more commonly used to study spinal degeneration (25, 28, 29). Additionally, most studies have measured elemental concentrations in bone tissue due to its ability to accumulate elements, enabling inferences about systemic changes in their profiles (30, 31). In contrast, elemental concentrations measured in serum, urine, or cerebrospinal fluid correspond to their current concentrations in these bodily fluids (30, 31). Therefore, the research conducted as part of this study is particularly valuable. Furthermore, it should be noted that the assessment of micro- and macronutrient concentrations in human degenerated IVDs has not yet been described in the specific compartments of IVDs, such as the NP and AF, as en bloc sampling during neurosurgical procedures precludes compartmental identification. Only in postmortem samples would it be possible to separate IVD compartments (32). Statistical analysis revealed that in degenerated intervertebral discs, significantly higher concentrations compared to control samples were noted for zinc, magnesium, calcium, and phosphorus (*p* < 0.05). Furthermore, the results presented in this paper indicate that determining the concentrations of iron, manganese, sodium, and magnesium may be useful in differentiating the severity of degenerative changes in intervertebral discs according to the Pfirrmann scale. However, statistical analysis did not show that the concentrations of the assessed microelements and macroelements depend on the intensity of L/S spine pain (*p* > 0.05). This may result from the fact that pain intensity is declarative and subjective and depends, among other factors, on an individual pain threshold. Patients may have inaccurately assessed the intensity of pain, and objective evaluation was further hindered by the imprecise visual analog scale.

Zn is a microelement involved in the metabolic processes of lipids, proteins, and carbohydrates; it also exhibits protective action against lead and cadmium poisoning, as well as excessive accumulation of free radicals, which play a significant role in the induction and development of oxidative stress—inseparably linked to the degenerative process (33). Its deficiency causes ailments of the muscular, nervous, digestive, reproductive, hormonal, immune, and skeletal systems (33). In this paper, significantly higher Zn concentrations were demonstrated in the analyzed samples compared to control samples (*p* < 0.05). Meanwhile, Mahmood et al. (34) and Grennan et al. (35) recorded significantly lower Zn concentrations in the serum of patients diagnosed with osteoarthritis of the knee compared to healthy volunteers (34, 35). These discrepancies may result from the use of different clinical materials for determining zinc concentration by me and the aforementioned researchers. Nonetheless, it should be remembered that Zn, just like Ca, is a cofactor of matrix metalloproteinases; thus, the increased concentration of this microelement in the analyzed samples indicates the presence of an active degenerative process and progressive degradation of the extracellular matrix of IVDs (36). The highest Zn concentration was observed in intervertebral disc samples representing the fourth degree of degeneration according to the Pfirrmann scale, while the lowest was noted in intervertebral discs with a degeneration level corresponding to the fifth degree of advancement. This suggests that in intervertebral discs with the highest degree of degeneration, the level of dehydration is so significant that it disrupts the transport of nutrients, thereby impairing the metabolic and biochemical processes in which enzymes participate (6). Moreover, statistical analysis revealed that Zn concentration differed significantly between the control group of women and men, from whom the intervertebral discs were obtained postmortem (*p* < 0.05).

Mg concentration also differed significantly between the analyzed and control samples (*p* < 0.05) and according to the degree of degeneration assessed using the Pfirrmann scale (*p* < 0.05). The highest Mg concentration was observed in intervertebral disc samples representing the second and fifth degrees of degeneration, while the lowest concentrations were noted in the third and fourth degrees of degeneration.

Mg has osteogenic properties and is a cofactor for nearly 300 enzymatic proteins, as well as an antagonist of calcium channels (37, 38). As such, magnesium is essential for maintaining proper neuromuscular conduction (37, 38); its deficiency manifests as fatigue, painful muscle spasms, tremors, numbness, and tingling in the extremities (37, 38). Zeng et al. (39) and Zhang et al. (40) demonstrated that magnesium concentration in the serum of patients with osteoarthritis of the knee is inversely proportional to the degree of degenerative changes within the cartilage. Additionally, Mg is suggested to have an analgesic effect in patients with degenerative spinal disease coexisting with neuropathic pain (41). Yousef and Al-deeb (41), evaluating the effectiveness of magnesium administration in reducing pain intensity, included patients who were already taking anticonvulsants and antidepressants before the study. Furthermore, non-steroidal anti-inflammatory drugs were used throughout the experiment (41).

Thus, the significantly higher Mg concentration observed in the analyzed samples compared to controls in this study highlights the crucial role of this macroelement in the calcification process of intervertebral discs. This conclusion appears even more justified given that the majority of magnesium-−60%—is found in the skeletal system in the form of hydroxyapatite crystals (38). Considering the results obtained for Mg concentration in degenerated IVDs depending on the radiological degree of advancement, it can be hypothesized that the osteogenic action of magnesium is intensified during the initial and advanced stages of degenerative disc disease in the L/S region.

The last two elements with significantly higher concentrations in degenerated IVDs compared to control samples were Ca and P. Together with vitamin D3, they are key regulators of hormonal balance and the skeletal and joint systems and ensure proper nerve impulse transmission (42).

In this research, higher calcium and phosphorus concentrations were noted in L/S IVDs affected by the degenerative process compared to control samples (*p* < 0.05). However, statistical tests did not confirm that the concentration profiles of calcium and phosphorus are useful for differentiating the degree of degenerative changes (*p* > 0.05).

Ninety-nine percent of the body's Ca is present in bones and teeth, with only 1% in body fluids in the ionized form (42). The results for Ca concentration in intervertebral discs are inconsistent and sometimes contradictory. For example, Chanchairujira et al. (43) reported the presence of calcium deposits in only 469 out of 3,568 postmortem IVDs, which constitutes 13% of the entire study group (43). In contrast, Feinberg et al. (44), who also used high-contrast radiography, observed calcium deposits in 34.5% of the intervertebral discs analyzed (44). Studies by Karamouzian et al. (45) confirmed a significantly higher incidence of Ca salt deposits in degenerated intervertebral discs compared to normal discs (54.4% vs. 6.7%; *p* < 0.05) (45). Gruber et al. (46), however, observed calcium deposits in only 14.7% of IVDs obtained during microdiscectomy (46). Similarly, Pytel et al. (47) reported a low percentage of degenerated IVDs (2.8%) with visible Ca salt deposits (47).

However, it should be emphasized that in this paper, calcium and other element concentrations were assessed using mass spectrometry techniques. This method enabled the quantitative assessment of total calcium in the L/S IVDs. The significance of abnormal Ca concentrations in the course of degenerative disc disease is highlighted by the studies of Stigen et al. (48), who used an animal model (dachshunds) to demonstrate that even when neuroimaging does not reveal IVD calcifications, the presence of calcium deposits can be confirmed histopathologically (48). Grant et al. (49) also showed that calcium concentration increased with the progression of disc degeneration assessed on the Thompson scale. Unfortunately, this study was conducted on only eight participants (49). Illien-Jünger et al. (50) confirmed Grant et al.'s (49) observations, emphasizing the occurrence of ectopic calcifications in IVDs in the context of advancing degenerative disc disease of the L/S region (50).

P, on the other hand, is the second dominant component of bone mass, present in bones and teeth as hydroxyapatite crystals or phosphoproteins. The significance of p in bone metabolism, including its role in degenerative diseases, was demonstrated in a study by Katsumata et al. (51), who found that rats fed a diet rich in phosphates exhibited increased concentrations of parathyroid hormone and bone turnover markers (51). Correlation analysis revealed a strong, statistically significant positive relationship between Ca and *p* concentrations in degenerated IVDs, underscoring the importance of changes in their concentrations for the progression of degenerative disc disease in the L/S region (*p* < 0.05).

Fe is a trace element that plays a significant role in the degenerative processes of cartilage and bone tissue as it serves as a cofactor for 25-hydroxycholecalciferol hydroxylase, an enzyme involved in the hydroxylation of lysine and proline residues during collagen fiber biosynthesis (52). Thus, considering Fe's role in collagen biosynthesis, one might expect its concentration to be lower in degenerated IVDs compared to control samples. However, this paper found higher iron concentrations in the analyzed samples compared to control samples (*p* < 0.05).

This observation might be due to the fact that Fe concentrations are higher in postmitotic tissues and increase with age, while the average age of the patients in the study group was higher than that of the control group members (53). Additionally, the observed profile of Fe concentrations in the analyzed and control samples might suggest intensified biosynthesis of collagen and elastin fibers in the degenerative process of IVDs as a mechanism of adaptation to changes in the extracellular matrix structure (6, 54). Similarly, Nganvongpanit et al. (53) demonstrated significantly higher Fe concentrations in the synovial fluid of degenerative knee joints, indicating that changes in the concentration of this trace element might serve as a useful marker for assessing the advancement of degenerative changes in the knee joint. This finding aligns with the analyses conducted as part of this article (53). Moreover, considering the radiological advancement of degenerative changes in the L/S IVDs, it seems that Fe primarily participates in the initial stages of the degenerative process. Its role appears to diminish in more advanced stages, likely due to decreased hydration of the IVDs (6, 55).

Although the statistical analysis did not reveal significant differences in the concentrations of Cu, Na, K, Pb, and Mn between the analyzed intervertebral discs and the control samples (*p* > 0.05), these elements are relevant in the context of bone turnover and degenerative changes in IVDs.

Cu is essential for the synthesis of heme and melanin and is a cofactor for several enzymes, including superoxide dismutase and lysyl oxidase, which are involved in collagen fiber crosslinking and bond formation (56). Cu deficiency leads to developmental bone abnormalities and an increased risk of osteoporosis or pathological fractures (56). Beyond its structural and enzymatic roles, recent research has identified a novel mechanism of copper-induced regulated cell death, termed cuproptosis, which is increasingly recognized as relevant in musculoskeletal pathologies. A recent study suggests that dysregulated copper homeostasis and cuproptosis-related pathways may contribute to tissue degeneration and remodeling in bone and cartilage disorders, raising the possibility of similar involvement in intervertebral disc degeneration (57). This highlights the need to further investigate copper-mediated cell death mechanisms in disc cells, as they may represent a new link between trace element imbalance and cellular loss during degeneration. The results obtained in this study differ from those presented by Kubaszewski et al. (58), who observed higher copper concentrations in IVDs in the second and third degrees of degeneration (58), as well as Dabrowski et al. (59), who reported higher Cu concentrations in degenerative hip joints (59). These differences could stem from the fact that the study group patients, who qualified for planned microdiscectomy, had been undergoing long-term conservative treatment with non-steroidal anti-inflammatory and analgesic drugs, muscle relaxants, and physical rehabilitation. This treatment might have significantly inhibited lipid peroxidation processes and lowered the concentration of ceruloplasmin, a protein capable of binding copper ions and whose levels increase during inflammatory states (60).

The next two elements studied were Na and K, which regulate Ca metabolism. Martini et al. (61) observed that for every 2.3 g of Na intake exceeding the recommended amount, an additional 24–40 mg of Ca is excreted in the urine (61). Prolonged excessive Na consumption results in Ca loss from bone tissue, decreased bone density, and consequently greater susceptibility to fractures and osteoporosis risk (61). Zhang et al. (62) evaluated Na, K, Fe, and Ca concentrations in the serum of 120 patients with degenerative L/S IVD disease compared to 24 healthy control participants. These authors did not report significant differences in Na and K concentrations between the study and control groups (*p* > 0.05) (62). However, they noted significantly reduced Ca and Fe concentrations in the serum of patients compared to the control group, with both decreasing as the radiological advancement of degenerative changes increased (62).

Erndt-Marino et al. demonstrated the positive effects of administering hyperosmolar K solutions for osteoarthritis of the knee (63). K is also linked to the occurrence of lumbosacral spine pain due to its critical role as a key mediator of neuropathic pain, regulated through ATP-sensitive potassium (KATP) channels located in primary afferent fibers and the spinal cord (64, 65).

K also regulates Ca concentrations, as higher K levels reduce calcium excretion in the urine (61). Thus, K protects bone tissue by reducing bone resorption and positively influencing bone mineral density (61). Furthermore, K is the body's main intracellular cation responsible for generating resting and action potentials in nerve cells, as well as maintaining water balance and acid–base equilibrium (66). Disruptions in Na and K concentrations are associated with abnormal K ion transport among the intracellular, extracellular, and intercellular spaces (66). Consequently, the observed decrease in K concentration in degenerative IVDs compared to healthy ones in this dissertation could result from IVDs dehydration. Studies show that potassium efflux from the intracellular space, combined with calcium ion influx, increases reactive oxygen species levels, which play a crucial role in inflammation induction and progression (67).

Mn is an essential trace element crucial for the development and function of the immune and nervous systems (68). It also regulates carbohydrate, lipid, and calcium metabolism and participates in the body's antioxidative processes (68). The studies conducted as part of this dissertation did not demonstrate significant differences in Mn concentrations between the analyzed and control samples (*p* > 0.05). Research on Mn levels in patients with degenerative spinal diseases has yielded mixed results. Mahmood (34) showed significantly lower Mn concentrations in the serum of patients with degenerative IVD disease compared to the control group (34). However, Zhang et al. (69) suggest that direct administration of drugs, peptides, and proteins encapsulated in manganese dioxide microspheres (MnO_2_) to degenerative intervertebral discs produced better therapeutic effects (including pain relief) than direct substance administration (69). Therefore, Mn appears to play an important role in the induction and progression of degenerative L/S disc disease, although the studies conducted here did not confirm this hypothesis.

Pb, the last element studied, exerts both direct and indirect effects on bone metabolism and turnover (70, 71). Directly, Pb decreases bone density and disrupts osteoblast and osteoclast metabolism, increasing fracture risk (70). Indirect effects include decreased plasma levels of 1,25-dihydroxycholecalciferol and parathyroid hormone (PTH) (70). Approximately 92% of lead in the body accumulates in the bones, and health consequences of Pb exposure are tied to its blood concentration (72). Blood Pb levels exceeding safety thresholds indicate severe lead poisoning (72). Patients in the study group reported being unaware of unintentional exposure to heavy metals. Filon et al. (73) and Kot et al. (74) detected lead concentrations exceeding permissible levels in certain grains, rice, and cereal products (73, 74). Considering lead accumulates in tissues over a lifetime, elevated concentrations may predispose individuals to lumbar-sacral degenerative disc disease.

Furthermore, the statistical analysis revealed that Mg, Ca, P concentrations vary significantly depending on BMI. Mg concentrations were highest in intervertebral disc samples from patients with normal BMI and lowest in those from obese patients. Additionally, vitamin D_3_ deficiencies are frequently observed in obese individuals (49, 52), and magnesium is essential for both the synthesis and activation of vitamin D_3_ (75). A randomized controlled trial suggests that maintaining optimal magnesium levels is fundamental for optimizing vitamin D3 concentrations (76).

Conversely, calcium and phosphorus concentrations were highest in intervertebral discs collected from obese patients and lowest in samples from individuals with normal BMI. Considering that calcium-rich diets or calcium supplementation contribute to weight loss and reduced white adipose tissue volume, the results confirm that the degenerative process in lumbar-sacral intervertebral discs is dynamic. It involves the simultaneous occurrence of two opposing processes: degeneration and regeneration, aimed at achieving homeostasis and driven by adaptive mechanisms responding to progressive degeneration (77).

Significant and strong correlations were confirmed between the concentrations of the analyzed micro- and macroelements in degenerative and healthy intervertebral discs. In the degenerative samples, significant relationships were found between potassium and manganese concentrations, as well as between calcium and phosphorus concentrations. In the control samples, significant correlations were noted between magnesium and copper, magnesium and manganese, and manganese and copper concentrations. These findings suggest the parallel occurrence of two opposing processes in lumbar-sacral intervertebral disc disease: degeneration and regeneration, both contributing to the pursuit of homeostasis.

The comparative analysis of oxidative stress biomarkers revealed marked redox disturbances in degenerated IVD tissues (14). TBARs levels, a key index of lipid peroxidation, were significantly elevated in the study group compared to controls (5.52 ± 2.52 vs. 2.63 ± 0.97; *p* < 0.0001), indicating enhanced oxidative membrane damage in degenerated discs. This observation is consistent with prior findings by Morales et al., who also reported significantly higher TBARs levels in lumbar disc degeneration (LDD) tissues (5.18 ± 4.14 vs. 2.56 ± 1.23; *p* = 0.008), reinforcing the link between disc degeneration and increased lipid peroxidation (78).

Sex-stratified analysis revealed a more pronounced TBARs elevation in females (*p* = 0.0255), aligning with the hypothesis that hormonal and metabolic factors may modulate oxidative burden in IVD pathology.

Although GSH levels were reduced in degenerated IVDs, the difference was marginally non-significant (*p* = 0.0539), suggesting early depletion of this primary non-enzymatic antioxidant during redox imbalance (79). This trend diverges slightly from Morales et al., who found no statistically significant differences in GSH between degenerated and non-degenerated groups (*p* = 0.708), possibly due to methodological or demographic differences (78).

Interestingly, GPx activity—a key enzymatic antioxidant—was significantly elevated in the study group (69.45 ± 3.92 vs. 60.25 ± 3.52; *p* = 0.0267), particularly in females, potentially reflecting an adaptive upregulation of antioxidant defenses in response to sustained oxidative stress (80).

BMI-stratified data showed that TBARs were most elevated in overweight individuals, whereas the highest GPx activity was recorded in those with normal BMI. This pattern implies that moderate adiposity may exacerbate oxidative processes, while normal-weight individuals may maintain a more efficient enzymatic antioxidant response. GSH levels were slightly higher in obese patients, which could represent a compensatory mechanism against chronic oxidative insults, though these differences did not reach statistical significance (81, 82).

Further stratification by Pfirrmann degeneration grade and VAS pain scores uncovered a progressive rise in TBARs and a concurrent decline in GSH, supporting a direct correlation between oxidative stress and structural degeneration. GPx activity demonstrated a non-linear trend, with decreased values from grade 2–4 followed by a slight rebound in grade 5 (*p* = 0.0410), which may indicate delayed enzymatic compensation in advanced degeneration. Similarly, a stepwise increase in TBARs and decrease in GSH across increasing VAS scores further validates the association between oxidative imbalance and subjective pain severity. Notably, GPx levels remained relatively stable across pain levels, suggesting a threshold beyond which enzymatic antioxidant systems may become saturated or ineffective.

Collectively, these results underscore oxidative stress as a central contributor to IVD degeneration and pain, marked by lipid peroxidation, glutathione depletion, and variably modulated GPx activity. These findings not only corroborate prior research but also provide new stratified insights, highlighting sex-, BMI-, and pain-related variability in redox dynamics. The potential for oxidative stress markers to serve as diagnostic indicators or therapeutic targets in disc degeneration merits further investigation, particularly in longitudinal and interventional contexts (83–85).

This study has several limitations that should be taken into account when interpreting the findings. First, the unequal size of the study and control cohorts, with 200 surgical specimens compared to 100 postmortem samples, may affect the statistical power of the analyses. The reliance on postmortem IVDs as controls, although unavoidable due to the lack of access to non-degenerated surgical material, introduces potential variability. Postmortem changes, including early autolysis and disturbances in water–electrolyte balance, could theoretically alter the stability of nutrients and oxidative stress markers. To minimize these effects, control tissues were collected within 48 h of death and verified histologically to exclude degenerative changes. Importantly, storage at low temperatures serves as a protective factor for biological material and is routinely applied in molecular biology to preserve molecular integrity. Furthermore, as highlighted by Zhang et al. (86), it is the degenerative cascade itself that leads to depletion of viable disc cells, thereby limiting the pool of cells available for protein extraction. From a methodological perspective, postmortem sampling remains the only feasible way to obtain non-degenerated IVDs, and similar approaches have been adopted in previous studies (87, 88).

Another limitation concerns the subjective nature of pain assessment using the VAS. Individual differences in pain thresholds, as well as the declarative character of self-reported data, may reduce the accuracy of these measurements. Finally, the en bloc sampling technique used in this study precluded separate evaluation of the nucleus pulposus and annulus fibrosus, limiting compartment-specific analysis.

In the present study, relatively large standard deviations were observed for some elemental concentrations, which might initially suggest potential outliers or data skewness. However, normality testing with the Shapiro–Wilk test confirmed that the data met parametric assumptions (*p* > 0.05), and no extreme values exerting undue influence were detected. Therefore, the observed variability likely reflects genuine inter-individual differences in metabolic status, body composition, and disease-related biochemical alterations rather than methodological artifacts. This natural biological heterogeneity strengthens the relevance of our findings, as it mirrors the variability encountered in clinical practice and highlights the complex interplay between trace element balance and IVD degeneration.

A critical limitation of our study is the substantial age difference between the surgical (mean age 49.6 ± 15.2 years) and postmortem control groups (mean age 37.3 ± 1.8 years). Age is among the strongest determinants of disc biochemistry, with progressive alterations in water content, extracellular matrix organization, and oxidative balance occurring even in the absence of overt degeneration. Numerous studies have demonstrated that age-related changes alone can influence trace element deposition and redox homeostasis, thereby confounding the interpretation of whether the differences observed in our cohort reflect primarily degenerative processes or, at least in part, normal aging physiology (14, 18, 30, 52). Unfortunately, age-matched non-degenerated surgical samples are not available, as non-degenerated IVDs are not removed for therapeutic reasons, and reliance on postmortem controls is an unavoidable methodological compromise also adopted in another IVD research (87, 88). Nevertheless, it must be acknowledged that the age disparity limits the ability to fully disentangle degeneration-related from age-related effects.

Despite these constraints, the study provides novel insights into the biochemical alterations underlying intervertebral disc degeneration and offers a solid basis for future research. This study provides valuable insights into the role of micro- and macronutrient concentrations in the degenerative processes of L/S IVDs. Significantly elevated levels of Zn, Mg, Ca, and P were observed in degenerated discs compared to non-degenerated controls, highlighting their potential roles in extracellular matrix degradation and oxidative stress regulation. While these findings suggest that nutrient imbalances may contribute to the degenerative process, they also emphasize the dynamic nature of this condition, involving concurrent degeneration and regeneration as adaptive mechanisms to achieve homeostasis. Variations in Fe, Mn, Na, and Mg concentrations were associated with the severity of degeneration as assessed by the Pfirrmann scale, suggesting their potential as biomarkers for disease progression. Future investigations should aim to include age-matched cohorts, larger postmortem series, or apply statistical modeling approaches to better disentangle degeneration-related from age-related changes in IVD biochemistry. In addition, longitudinal studies examining systemic correlations and the therapeutic potential of nutrient modulation may further clarify causal pathways and inform strategies to mitigate degenerative IVD disease.

## Data Availability

The raw data supporting the conclusions of this article will be made available by the authors, without undue reservation.
